# Identification of Factors Associated with Mortality in the Elderly Population with SARS-CoV-2 Infection: Results from a Longitudinal Observational Study from Romania

**DOI:** 10.3390/ph17020202

**Published:** 2024-02-03

**Authors:** Victoria Birlutiu, Bogdan Neamtu, Rares-Mircea Birlutiu

**Affiliations:** 1 Faculty of Medicine, Lucian Blaga University of Sibiu, Str. Lucian Blaga, Nr. 2A, 550169 Sibiu, Romania; victoria.birlutiu@ulbsibiu.ro (V.B.); bogdan.neamtu@ulbsibiu.ro (B.N.); 2 County Clinical Emergency Hospital, Bvd. Corneliu Coposu, Nr. 2-4, 550245 Sibiu, Romania; 3 Pediatric Research Department, Pediatric Clinical Hospital Sibiu, Str. Pompeiu Onofreiu, Nr. 2-4, 550166 Sibiu, Romania; 4 Faculty of Medicine, University of Medicine and Pharmacy “Carol Davila”, 050474 Bucharest, Romania; 5 Clinical Hospital of Orthopedics, Traumatology, and Osteoarticular TB Bucharest, B-dul Ferdinand 35-37, Sector 2, 021382 Bucharest, Romania

**Keywords:** SARS-CoV-2, COVID-19, elderly population, risk factors, mortality

## Abstract

The progression of SARS-CoV-2 infection has been linked to a hospitalization rate of 20%. The susceptibility of SARS-CoV-2 infection increases with age, resulting in severe and atypical clinical forms of the disease. The severity of SARS-CoV-2 infection in the elderly population can be attributed to several factors, including the overexpression of angiotensin-converting enzyme 2 (ACE2) receptors, immunosenescence, and alterations in the intestinal microbiota that facilitate the cytokine storm. In light of these observations, we conducted a retrospective analysis based on prospectively collected data between 23 December 2021 and 30 April 2022 (the fourth wave of SARS-CoV-2 infection). We analyzed patients aged over 60 years who were hospitalized in a county hospital in Romania. The primary objective of our study was to assess the risk factors for an unfavorable outcome, while the secondary objective was to assess the clinical and baseline characteristics of the enrolled patients. We included 287 cases with a complete electronic medical record from this available cohort of patients. We aimed to retrospectively evaluate a group of 127 patients that progressed, unfortunately, toward an unfavorable outcome versus 160 patients with a favorable outcome. We used the Combined Ordinal Scale of Severity that combines the WHO ordinal scale and the degrees of inflammation to assess the severity of the patients at the time of the initial assessment. The age group between 70 and 79 years had the highest percentage, accounting for 48.0%—61 patients, of the deceased patients. We noted statistically significant differences between groups related to other cardiovascular diseases, nutritional status, hematological diseases, other neurological/mental or digestive disorders, and other comorbidities. Regarding the nutritional status of the patients, there was a statistically significant unfavorable outcome for all the age groups and the patients with a BMI > 30 kg/m^2^, *p* = 0.004. The presence of these factors was associated with an unfavorable outcome. Our results indicate that with the presence of cough, there was a statistically significant favorable outcome in the age group over 80 years, *p* ≤ 0.049. In terms of the presence of dyspnea in all groups of patients, it was associated with an unfavorable outcome, *p* ≤ 0.001. In our study, we analyzed laboratory test results to assess the level of inflammation across various WHO categories, focusing on the outcome groups determined by the average values of specific biomarkers. Our findings show that, with the exception of IL-6, all other biomarkers tend to rise progressively with the severity of the disease. Moreover, these biomarkers are significantly higher in patients experiencing adverse outcomes. The differences among severity categories and the outcome group are highly significant (*p*-values < 0.001). CART algorithm revealed a specific cut-off point for the WHO ordinal scale of 4 to stand out as an important reference value for patients at a high risk of developing critical forms of COVID-19. The high death rate can be attributed to proinflammatory status, hormonal changes, nutritional and vitamin D deficiencies, comorbidities, and atypical clinical pictures.

## 1. Introduction

The SARS-CoV-2 infection’s evolution has been observed to be associated with a hospitalization rate of 20% [1]. A progression to the critical stage was reported in 5% of the hospitalized cases, with the development of acute respiratory distress syndrome (ARDS), sepsis, and multiple system organ failure (MSOF) [1,2].

The patient’s advanced age and comorbidities, including high blood pressure, diabetes, male gender, obesity, immunosuppression, chronic kidney disease or respiratory disorders, and other cardiovascular and neurological disorders, are significant factors that can contribute to the worsening of the clinical evolution and outcome of SARS-CoV-2 infection. Infection with the Delta variant (B.1.617.2) of the SARS-CoV-2 virus requires supplemental oxygen therapy in 75% of hospitalized cases [3].

Susceptibility of SARS-CoV-2 infection increases with age and so does the probability of presenting with severe and atypical clinical forms of the disease. These unique presentations are often associated with neurological or psychiatric manifestations, such as strokes, ischemic or hemorrhagic strokes, hallucinations, altered mental status, delirium, and syncope [4]. In a local study on 25 patients treated with off-label tocilizumab and adjuvant iron chelator in a group of severe COVID-19 pneumonia, the authors reported that among the deceased cases, 80% of patients had multiple (3 or 4) comorbidities, 60% were older than 60 years and had elevated levels of serum glucose (80%) (>125 mg/dL) and ferritin (60% above 1000 ng/mL) [5].

Pneumomediastinum is a rare complication of acute respiratory distress syndrome (ARDS). However, during the COVID-19 pandemic, it has become more common. In a recent study, researchers found that COVID-19 patients with pneumomediastinum showed a unique fibrous hyaline degeneration of the tracheal rings, which has not been observed before [6]. Also, it has been reported that both mechanical and spontaneous ventilation-related pneumomediastinum were described in COVID-19 patients. While the cause of pneumomediastinum in COVID-19 patients is not fully understood, some experts suggest that it may be related to cartilage remodeling during ARDS, without a complete post-injury regenerative process [6]. This remodeling process can lead to tracheal and large airway injury, which is a pathologic signature observed in COVID-19 patients with pneumomediastinum. The median (interquartile range) age of patients with pneumomediastinum was 73 (64–77) years in the study of Baratella et al. [6].

Neurotropic properties and cutaneous manifestations of SARS-CoV-2 are increasingly being recognized, also in the older population [7,8]. Results from an observational study on 80 cases of SARS-CoV-2-Clostridioides difficile co-infection showed that recovered patients present a statistically significant decreased median age than the patients who did not survive (67 vs. 76, *p* < 0.05, Mann–Whitney U test) [9].

With the emergence of COVID-19, researchers have been using various cell lines for in vitro models to study the SARS-CoV-2 virus and its implications on the disease. Among these cell lines, the Calu-3 cell line has proven to be one of the most valuable. Analysis of transcriptomes from SARS-CoV-2-infected cells has revealed cellular responses related to IFN signaling, innate immunity, inflammation, and defense against the virus. These transcriptional changes, even though temporary, can help characterize the early stages of the cellular response to SARS-CoV-2 infection, providing insights into COVID-19 pathogenesis at the airway epithelium level, as well as revealing potential biomarkers and drug targets [10].

Data from a recent observational study on acute kidney injury progression in COVID-19 patients report diabetes, dyspnea on admission, the need for supplemental oxygen, and admission to the intensive care unit as having a crucial role in producing unfavorable outcomes, with a death rate of more than 50%, in patients with a mean age of 67.55 years [11].

In the era of artificial intelligence (AI), Ippolito et al. recently published an article evaluating AI’s role in analyzing chest X-rays in the emergency department to aid in differentiating lung pneumonia. The study reveals that the AI system could be a valuable tool for radiologists in identifying COVID-19 pneumonia, particularly in emergency departments where quick diagnosis is critical. Its capability to recognize and distinguish different lung infection patterns could speed up clinical decision-making, enhancing the diagnosis of pulmonary diseases and shortening response times. Additionally, the AI system can accurately distinguish between COVID+, pneumonia+, and healthy individuals, boosting radiologists’ diagnostic accuracy and offering a highly efficient second opinion [12].

The severity of SARS-CoV-2 infection in the elderly population can be attributed to several factors, including the overexpression of angiotensin-converting enzyme 2 (ACE2) receptors, immunosenescence, and alterations in the intestinal microbiota that can facilitate the cytokine storm. Furthermore, sex hormone imbalances, particularly in females, as well as deficiencies in growth hormones that play a vital role in immunoregulation, contribute to the severity of the disease. Additionally, cellular changes, oxidative stress, and mitochondrial dysfunction are also key factors in the progression of COVID-19 in the elderly, as well as in the context of obesity [4].

The presence of SARS-CoV-2 in cerebrospinal fluid and neurons, glial cells, or neuroepithelium [13,14] is responsible for the occurrence of neurodegenerative lesions, hyperphosphorylation, as well as for the dislocation of the Tau protein [15,16]. Cathepsina L, furin, and Neuropilin-1 together with ACE2 receptors and transmembrane protein serine protease 2 (TMPRESS2) have been found to play a crucial role in neuronal invasion [17,18,19,20]. The previously mentioned mechanisms and proteins are often accompanied by cerebral hypoxia, pulmonary encephalopathy [19], coagulation, and microcirculation disorders, as well as inflammatory phenomena that can increase the damage caused by the virus in the central nervous system [21]. Additionally, autoimmune mechanisms may be triggered by the viral infection, leading to demyelination, multiple sclerosis, acute disseminated encephalopathy, optic neuritis, acute necrotizing encephalopathy, and other related complications [22,23].

The onset of anosmia or ageusia has been reported in around 50% of patients with SARS-CoV-2 infection [24,25]. These symptoms can occur early in the course of the infection and can even appear before the respiratory symptoms [24,25], being the first symptoms of the disease [26]. Depression in the elderly population and pre-existing cognitive disorders can exacerbate the progression of COVID-19 infection. Moreover, individuals suffering from dementia or Alzheimer’s disease have been found to have a higher mortality rate [27].

In elderly patients, the inflammation status that promotes the clarence of senescent cells leads to an increase in the levels of pro-inflammatory cytokines, such as IL-6, IL-1RA, TNF-α, and IL-1. This process also activates microglia, macrophages, and dendritic cells and increases the activity of NF-κB, cyclooxygenase-2 (COX-2), and inducible nitric oxide synthase (iNOS), which further release IL-6, IL-1, and TNF-α. In addition to these immune system changes, nutritional deficiencies and hypoalbuminemia also contribute to the severity of the SARS-CoV-2 infection in the elderly. The deficiency of 25-hydroxy vitamin D is a vulnerability factor in the elderly, especially in males, as it plays a crucial role in preventing cytokine storms through its immunomodulatory action [28].

Based on the previously mentioned data from the literature, we hypothesized that different factors are associated with mortality in the elderly population with SARS-CoV-2 infection. As a result, in carrying out this retrospective analysis of prospectively collected data from a single-center longitudinal observational cohort, we aimed to assess the risk factors for an unfavorable outcome at the time of discharge from the hospital and report the clinical and baseline characteristics of the enrolled patients.

## 2. Results

Of the hospitalized patients in our clinic between 23 December 2021 and 30 April 2022 (the fourth wave of SARS-CoV-2 infection), 366 were patients over 60 years old. We included 287 cases with a complete electronic medical record from this available cohort of patients. We aimed to retrospectively evaluate a group of 127 patients that progressed, unfortunately, toward an unfavorable outcome versus 160 patients with a favorable outcome.

The ages of the 287 patients ranged between a minimum of 60 and a maximum of 94 years, with a mean of 74.56 years and a standard deviation of ±7.40 years. The mean age of the 127 patients with an unfavorable outcome was 75.15 years and a standard deviation of 7.29 years, versus the 160 patients with a favorable outcome with a mean age of 74.12 years and a standard deviation of 8.41 years.

Of the 287 enrolled patients, 127 were male (44.3%). All 287 patients were distributed by age in three groups (60–69 years, 70–79 years, and 80 years or above).

The length of hospitalization of the 287 patients ranged between a minimum of 1 day and a maximum of 54 days, with a mean of 10.49 days and a standard deviation of ±6.52 days. The mean length of hospitalization of the 127 patients with an unfavorable outcome was 9.75 days and a standard deviation of 6.97 days, versus the 160 patients with a favorable outcome with a mean age of 11.09 days and a standard deviation of 6.10 days.

According to the COVID-19 pneumonia severity grade classification, 1 of the 127 enrolled patients with an unfavorable outcome had a mild and moderate form of the disease. About 16 patients were seriously ill, and 109 cases were critically ill. Regarding the patients with a favorable outcome, 16 patients had a mild form of the disease, 96 had a moderate one, 40 were serious, and 8 were critically ill.

In terms of oxygen requirements, for both groups, all patients required noninvasive ventilation (oxygen mask, continuous positive airway pressure, or high-flow nasal cannula oxygen), and just for the patients with unfavorable outcomes, invasive mechanical ventilation was also required. An overview of the enrolled patients is reported in Table 1.

A detailed overview of the comorbidities of the enrolled patients is highlighted in Table A1.

We noted statistically significant differences between groups related to other cardiovascular diseases, nutritional status, hematological diseases, other neurological/mental or digestive disorders, and other comorbidities (Table A2). An increased number of patients from the study group of patients with a favorable outcome was noticed to have the following comorbidities: other cardiovascular diseases, a BMI of 18.5–24.9 or 25–29.9 kg/m^2^, hematological diseases, other neurological/mental or digestive disorders, and other comorbidities (Table A2).

A cross-tabulation between comorbidities by outcome and age group was also performed. Regarding other cardiovascular comorbidities, there was a statistically significant favorable outcome in all three age groups, *p* ≤ 0.001 (Table A3). In terms of type 2 diabetes mellitus, there was a statistically significant favorable outcome for the patients in the age group 70–79 years, *p* ≤ 0.028 (Table A4). Regarding the nutritional status of the patients, there was a statistically significant unfavorable outcome for all the age groups and the patients with a BMI > 30 kg/m^2^, *p* = 0.004 (Table A5). Regarding hematological malignancies, there was a statistically significant favorable outcome for the age group 60–69 years and 70–79 years, *p* = 0.002 (Table A6). In terms of the presence of digestive disorders, there was a statistically significant favorable outcome for the patients in the age group (60–69 years), *p* ≤ 0.004 (Table A7). In terms of the presence of other comorbidities, there was a statistically significant favorable outcome for the patients in the age group 70–79 years, *p* ≤ 0.001 (Table A8).

A detailed overview of the clinical manifestations of the enrolled patient at the time of admission into the hospital is highlighted in Table A9. The dataset reveals valuable insights into the prevalence of various clinical manifestations within our study population at the time of admission. Firstly, concerning fever, 36.9% of patients reported experiencing it, while the majority, constituting 63.1%, did not exhibit this symptom. Regarding respiratory symptoms, cough was reported by 61% of patients, whereas 39% did not experience it. Similarly, dyspnea was present in 60.6% of cases. Headache was reported by 22.6% of patients, while lethargy was present in 30.3% of cases. Fatigue was reported by 57.1% of enrolled patients, and nausea, vomiting, and diarrhea were less prevalent, reported by 8.0%, 7.7%, and 7.7%, respectively. Other symptoms, such as abdominal pain, sore throat, anosmia, and ageusia, were reported by a minority of patients, ranging from 4.2% to 9.1%. Additionally, our dataset also includes information on less common symptoms such as the presence of viral exanthema, myalgia, arthralgias, diaphoresis, nasal congestion/rhinorrhea, epistaxis, chest pain/discomfort, acute neurological disorders (stroke, transient ischemic attack, and decompensation of a known neurological injury), and acute cardiac disorders (myocardial infarction and heart rhythm disorders with an acute onset—mainly atrial fibrillation).

The studied dataset highlights the correlation between various clinical manifestations at admission and the clinical outcomes within the studied cohort, employing Fisher’s exact test for rigorous statistical evaluation. The binary outcomes, namely, “Deceased” and “Alive,” were examined in conjunction with specific symptoms, and the ensuing *p*-values from Fisher’s exact test indicate the statistical significance of the observed associations. Focusing on the manifestation of fever, our data reveal no statistically significant association with outcomes (*p* = 0.902). Similarly, the presence or absence of cough demonstrates a borderline statistical association with outcomes (*p* = 0.051), hinting at a potential connection between cough and the dichotomous outcomes of mortality or a favorable outcome (alive). Conversely, dyspnea emerges as a significant factor associated with outcomes (*p* < 0.001), substantiating its potential prognostic relevance. However, headache, lethargy, nausea, vomiting, and diarrhea do not exhibit statistically significant associations with outcomes (*p*-values ranging from 0.119 to 0.438).

Regarding fatigue, it also stands out as significantly associated with the outcome (*p* < 0.001), suggesting a potential prognostic role in mortality. Notably, abdominal pain demonstrates a trend toward significance (*p* = 0.084), indicating a potential but less conclusive association. Furthermore, symptoms such as sore throat, anosmia, and ageusia exhibit statistically significant associations with outcomes (*p*-values ranging from 0.002 to 0.020), implying their potential relevance as prognostic indicators in the context of mortality. The dataset also encompasses additional symptoms, including myalgia, arthralgias, diaphoresis, nasal congestion/rhinorrhea, epistaxis, chest pain/discomfort, acute neurological, and acute cardiac disorders (Table 2).

A cross-tabulation between symptoms by outcome and age group revealed the following results. Regarding cough, there was a statistically significant favorable outcome in the age group over 80 years, *p* ≤ 0.049 (Table A10). In terms of the presence of dyspnea in all groups of patients, it was associated with an unfavorable outcome, *p* ≤ 0.001 (Table A11). The presence of headaches in patients in the age group >80 years was associated with a favorable outcome, *p* = 0.001 (Table A12). Fatigue in patients in the age group >80 years was associated with a favorable outcome, *p* = 0.005 (Table A13). The presence of sore throat in the age group 60–69 years was associated with a favorable outcome, *p* = 0.014 (Table A14).

A detailed insight into the distribution and variability of the main studied laboratory parameters and imaging diagnostic scores within the studied population was performed and summarized in Table 3. Urea levels varied from 12.00 mg/dL to 427.00 mg/dL, with a mean of 67.19 mg/dL and a standard deviation of 56.20 mg/dL. Creatinine levels ranged from 0.57 mg/dL to 13.22 mg/dL, with a mean of 1.94 mg/dL and a standard deviation of 2.09 mg/dL. Alanine transaminase exhibited a minimum of 8.00 U/L and a maximum of 8004.00 U/L, with a mean of 82.80 U/L and a standard deviation of 494.97 U/L. Aspartate transferase values ranged from 6.00 U/L to 5887.00 U/L, with a mean of 63.95 U/L and a standard deviation of 366.53 U/L. Sodium levels varied from 121 mEq/L to 154 mEq/L, with a mean of 138.61 mEq/L and a standard deviation of 4.42 mEq/L. Potassium levels ranged from 2.39 mEq/L to 8.12 mEq/L, with a mean of 4.28 mEq/L and a standard deviation of 0.92 mEq/L. Ferritin levels exhibited a minimum of 9.00 mg/mL and a maximum of 3375.40 mg/mL, with a mean of 569.71 mg/mL and a standard deviation of 604.27 mg/mL. D-Dimers ranged from 133.60 ng/mL to 50,000.00 ng/mL, with a mean of 4417.15 ng/mL and a standard deviation of 9430.71 ng/mL. Creatine phosphokinase levels varied from 13 to 9468 IU/L, with a mean of 370.98 IU/L and a standard deviation of 963.71 IU/L. Lactate dehydrogenase levels ranged from 108 U/L to 7199 U/L, with a mean of 464.25 U/L and a standard deviation of 557.984 U/L. IL-6 levels varied from 4.04 pg/mL to 710.10 pg/mL, with a mean of 87.98 pg/mL and a standard deviation of 132.06 pg/mL. C-reactive protein levels ranged from 0.47 mg/dL to 557.00 mg/dL, with a mean of 96.71 mg/dL and a standard deviation of 96.64 mg/dL. Fibrinogen levels exhibited a minimum of 81 mg/dL and a maximum of 1274 mg/dL, with a mean of 497.38 mg/dL and a standard deviation of 199.35 mg/dL. Erythrocyte sedimentation rate ranged from 1 mm/h to 120 mm/h, with a mean of 38.82 mm/h and a standard deviation of 28.20 mm/h. White blood cell counts varied from 13/µL to 66,700/µL, with a mean of 16,289.66/µL and a standard deviation of 15,015.44/µL. The neutrophil-to-lymphocyte ratio ranged from 0.10 to 90.73, with a mean of 11.96 and a standard deviation of 12.53. The pulmonary CT-scan score ranged from 0 to 25, with a mean of 10.40 and a standard deviation of 8.50.

A cross-tabulation analysis between laboratory parameters and imaging diagnostic scores within the studied population and outcome was also performed and revealed statistically significant differences between groups, with higher mean values (of urea, creatinine, alanine transaminase, D-dimers, creatine phosphokinase, lactate dehydrogenase, C-reactive protein, fibrinogen, erythrocyte sedimentation rate, WBCs, neutrophil-to-lymphocyte ratio, and pulmonary CT-scan score) in the group with an unfavorable outcome with *p*-values ranging from 0.000 to 0.019. A summary of the data is reported in Table A15.

A cross-tabulation analysis between laboratory parameters and imaging diagnostic scores within the studied population by outcome and age of the patients by age group was also performed and revealed statistically significant differences between age groups, with higher mean values (of urea—age group >80 years, creatinine—age group >80 years, ferritin—age group 70–79 years, D-dimers—age group 60–69 years, creatine phosphokinase—age group 70–79 years, lactate dehydrogenase—age group 60–69 years, C-reactive protein—age group 60–69 years, fibrinogen—age group 70–79 years, erythrocyte sedimentation rate—age group 70–79 years, WBCs—age group 60–69 years, neutrophil-to-lymphocyte ratio—age group 70–79 years, and pulmonary CT-scan score—age group 70–79 years) in the group with an unfavorable outcome with *p*-values ranging from 0.000 to 0.019. A summary of the data is reported in Table A16.

**Figure 1:**
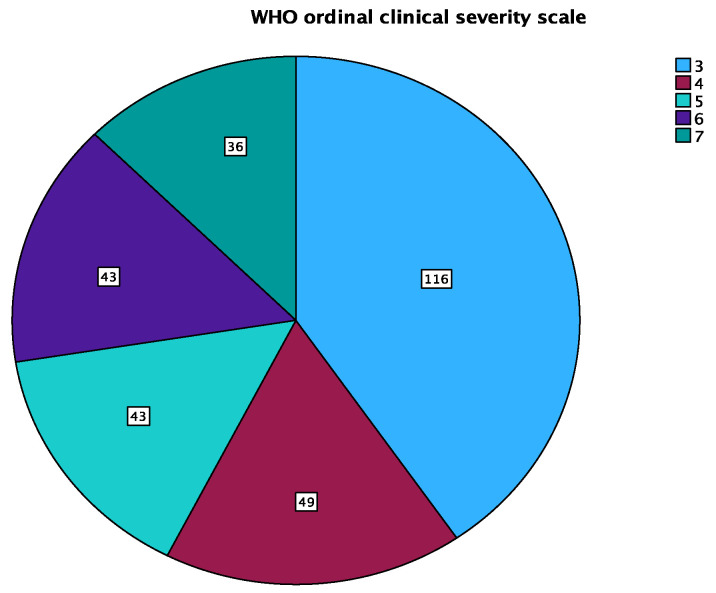
WHO ordinal clinical severity scale.

As this was a hospital-based study, the patients enrolled in this study corresponded to categories 3 to 7 of the WHO ordinal clinical severity scale. Figure 1 highlights the number of patients in each WHO ordinal clinical severity scale category. Table 4 illustrates the laboratory test results and the degree of inflammation between the different WHO categories. The table presents mean values of various biomarkers at different categories of severity based on the WHO ordinal clinical severity scale (categories 3 to 7). The mean D-dimer values increase progressively with the severity of the disease. The differences among severity categories are highly significant (*p* < 0.001). Ferritin levels also rise with the severity of the disease; the differences among severity categories are also highly significant (*p* < 0.001). Lactate dehydrogenase values follow a similar pattern. IL-6 values show less variation than the other studied inflammatory markers, and there is no significant difference among severity categories (*p* = 0.105). C-reactive protein levels increase with severity. The differences among severity categories are also highly significant (*p* < 0.001). The neutrophil-to-lymphocyte ratio rises with severity, and the differences among severity categories are highly significant (*p* < 0.001).

Table 5 illustrates the laboratory test results that evaluated the degree of inflammation between the different WHO categories according to the outcome group. The table presents mean values of the same biomarkers as in Table 4 for different categories of severity based on the WHO ordinal clinical severity scale. The mean values of the biomarkers except IL-6 increase progressively with the severity of the disease and are more increased for the patients with an unfavorable outcome. The differences among severity categories and the outcome group are highly significant (*p*-values < 0.001).

Table A17 outlines the distribution of patients across different risk categories of inflammation based on the WHO ordinal clinical severity scale. The risk categories are classified as low, intermediate, and high and cross-tabulated against the severity levels.

In the low-risk category, 82 patients are in severity category 3, 32 are in category 4, 8 are in category 5, and no individuals are in categories 6 and 7. The total number of individuals in the low-risk category is 122.

For the intermediate-risk category, there are 25 patients in severity category 3, 9 in category 4, 21 in category 5, 28 in category 6, and 23 in category 7. The total number of patients in the intermediate-risk category is 106.

In the high-risk category, there are 9 patients in severity category 3, 8 in category 4, 14 in category 5, 15 in category 6, and 13 in category 7. The total number of patients in the high-risk category is 59.

Table A18 highlights the conducted statistical assessment of a cross-tabulation between the WHO ordinal clinical severity scale, the risk categories of inflammation, and the outcome. The analysis unveils a nuanced, yet statistically non-significant, inclination towards increased mortality rates in tandem with heightened clinical severity, especially salient in patients classified under intermediate- and high-risk categories of inflammation (*p* = 0.090). While indicative of a potential correlation, this trend does not reach the statistical significance threshold, suggesting a complex interplay of factors influencing mortality that extends beyond the scope of inflammation risk alone. Conversely, in the favorable outcomes dataset (alive), most of the patients categorized as alive predominantly fall within the low-risk inflammation category. This tendency is particularly pronounced at the lower rungs of the clinical severity scale (levels 3 and 4).

Nevertheless, similar to the mortality trend, this observation does not attain statistical significance (*p* = 0.926), hinting at the multifactorial nature of a favorable outcome. However, a pivotal finding emerges when the outcomes of “Deceased” and “Alive” are synthesized into a comprehensive analysis. The statistical analysis reveals a statistically significant correlation (*p* < 0.001) across the various levels of clinical severity, underscoring a compelling linkage between the elevation in inflammation risk levels and an increase in mortality rates. This significant correlation indicates that as patients progress through higher levels of clinical severity, the risk category of inflammation becomes an increasingly influential factor in determining their outcome.

Table 6 summarizes a comprehensive statistical analysis correlating various comorbidities and other health factors with the WHO ordinal clinical severity scale. The analysis demonstrates a nuanced relationship between these health factors and the severity scale. Firstly, age appears to increase with severity slightly, but this trend is not statistically significant, with a *p*-value of 0.769. Gender, however, shows a significant variance across severity levels (*p* = 0.011), indicating a potential gender-based difference in clinical outcomes. Hypertension (defined, based on the guidelines provided by the American Heart Association (AHA) and the American College of Cardiology (ACC), as a systolic blood pressure (SBP) of 130 mm Hg or higher or diastolic blood pressure (DBP) of 80 mm Hg or higher), while a common health concern, does not correlate significantly with clinical severity (*p* = 0.314). Other cardiovascular diseases (previously known as heart rhythm disorders, coronary artery disease, cardiomyopathy, valvular heart disease, and heart failure) are significantly correlated with severity levels (*p* < 0.001). In contrast, type 2 diabetes mellitus (characterized by high blood sugar levels, insulin resistance, and relative deficit of insulin) does not correlate significantly (*p* = 0.122). Chronic kidney disease (was considered for any kidney disease condition resulting in the gradual loss of kidney function quantitatively assessed over time by the decline in the glomerular filtration rate (GFR) and concomitant with albuminuria) is another factor with a notable correlation (*p* = 0.014), indicating its relevance in determining clinical severity. As indicated by BMI, nutritional status shows significant differences across severity levels (*p* < 0.001). Furthermore, while hematological malignancy demonstrates a significant correlation with clinical severity (*p* < 0.001), solid tumors (gastrointestinal, bronchopulmonary, and prostate neoplasia) do not (*p* = 0.204). Other health factors such as stroke, neurological disorders, acute psychiatric pathology, and other mental disorders do not present significant correlations, suggesting a more complex interaction with the clinical severity scale.

**Figure 2:**
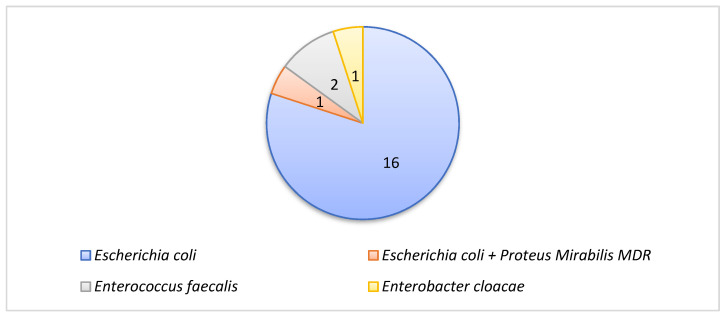
Bacterial strains isolated from urine cultures, number of strains.

**Figure 3:**
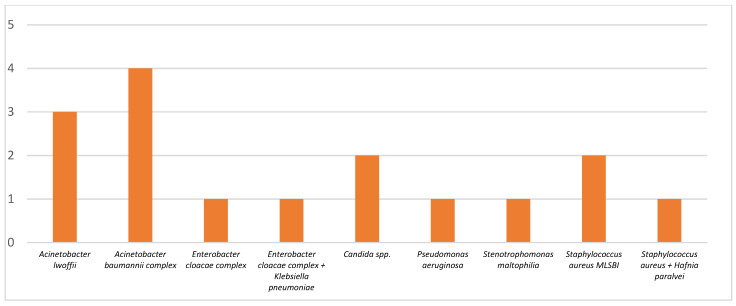
Bacterial strains isolated from tracheal aspirate cultures, number of strains.

Bacterial co-infections in the group of patients with an unfavorable outcome were represented by sepsis associated with positive blood cultures in 1 case (due to *Streptococcus sanguinis*), with urinary tract infections in 20 cases, and with positive cultures of the tracheal aspirate in 16 cases. With a positive culture from wound secretion samples in 3 cases, isolated strains are illustrated in Figure 2 and Figure 3.

Treatment for SARS-CoV-2 infection in this study adhered to the prevailing local and national guidelines. We administered Remdesivir to 112 patients, 76 of whom experienced favorable outcomes, and Favipiravir to 104 patients, with 44 reporting favorable outcomes. Remdesivir was administered as a loading dose of 200 mg IV over 30 to 120 min on day 1, followed by 100 mg IV qday for 5 days. In patients requiring invasive mechanical ventilation, treatment was extended to 10 days. Favipiravir was administered as a loading dose of 1.6 g q12h on day 1, followed by 600 mg q12h for 7 to 14 days. Immunomodulatory therapy was also integral to the treatment plan. Tocilizumab was administered to 13 out of 287 patients, with 6 reporting favorable outcomes. Anakinra was given to 34 patients, with 6 favorable outcomes. Glucocorticoids were prescribed to 184 patients, with 78 reporting favorable outcomes. The antibiotic therapy was varied: cephalosporins (135 patients, 52 favorable outcomes), carbapenems (61 patients, 6 favorable outcomes), macrolides (1 patient, unfavorable outcome), aminoglycosides (4 patients, 2 favorable outcomes), polypeptides (30 patients, 2 favorable outcomes), oxazolidinones (75 patients, 12 favorable outcomes), tetracyclines (38 patients, 4 favorable outcomes), fluoroquinolones (37 patients, 8 favorable outcomes), and glycopeptides (21 patients, 12 favorable outcomes). Antimycotics were prescribed in 93 cases, resulting in 22 favorable outcomes.

We then included all the predictors showing statistical significance in the univariate analysis; the logistic regression model was applied first with respect to the outcome (death/alive) and then the CART decision tree with clinical forms (moderate, severe, critical) as the dependent variable.

The binary logistic regression model oiutput was $\log\left(\frac{p}{1-p}\right)=-2.357+0.007\;\times \;$ LDH + 0.106 × NLY ratio, where *p* = 1 was the probability for death to occur.

For each unit increase in LDH, there was a 0.007 increase in log odds of the event occurring (odds ratio (OR), 1.007; 95% CI, 1.002–1.012) 

For each unit increase in the NLY ratio, there was a 0.106 increase in log odds of the event occurring (odds ratio (OR), 1.112; 95% CI, 1.025–1.206).

**Figure 4:**
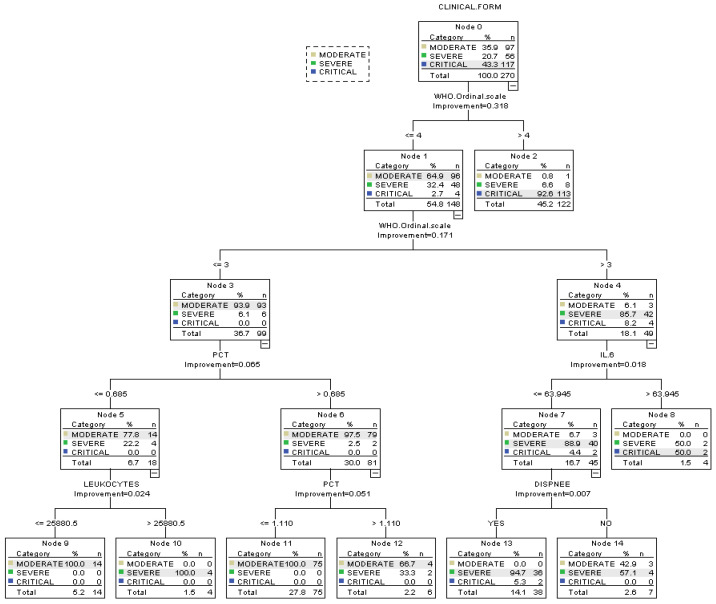
Decision path for each risk group.

Our CART algorithm approach yielded decision paths suggesting three categories at risk for severe and critical forms: low-risk (≤34%), medium-risk (>50%), and high-risk categories (>90%) and for moderate clinical form also three categories: low-risk (42.9%), medium-risk (66.7%), and high-risk categories (100%). Specific cut-off points emerged for each parameter as independent variables in the decision tree with significance to the outcome (Figure 4).

**High-risk group for severe and critical forms (>90%):**

- **Critical form:**
  ○ 113 patients WHO scale >4 (92.6%—Node 2)
- **Severe form:**
  ○ 36 patients with 3 ≤WHO scale <4, IL-6 < 63.95 pg/mL, Dyspnea—Yes (94.7%—Node 13)
  ○ 4 patients, WHO scale ≤3, PCT < 0.685 ng/mL, WBCs > 25,880 µL (100%—Node 10)

**Medium-risk group for severe and critical forms (>50%):**

- **Critical form (50%):**
  ○ 2 patients with 3 ≤WHO scale <4, IL-6 > 63.946 ng/mL (50%—Node 8)
- **Severe form (50%):**
  ○ 2 patients with 3 ≤WHO scale <4, IL-6 > 63.946 ng/mL (50%—Node 8)
  ○ 4 patients with 3 ≤WHO scale <4, IL-6 < 63.95 ng/mL, Dyspnea—No (57.1%—Node 14)

**Low-risk group for severe and critical forms (≤34%)**

- **Critical form:**
  ○ 2 patients with 3 ≤WHO scale ≤4, IL-6 < 63.95 pg/mL, Dyspnea—Yes (5.3%—Node 13)
- **Severe form:**
  ○ 2 patients with 3 ≤WHO scale ≤4, PCT > 1.11 ng/mL (33.3%—Node 12)

**Probability of a moderate clinical form:**

- **High (100%):**
  ○ 14 patients, WHO scale ≤3, PCT < 0.685 pg/mL, WBCs < 25,880.5 µL (100%—Node 9)
  ○ 75 patients, WHO scale ≤3, PCT ≤ 1.11 ng/mL (100%—Node 11)
- **Medium (66.7%):**
  ○ 4 patients, WHO scale ≤3, PCT > 1.11 ng/mL (66.7%—Node 12)
- **Low (42.9%):**
  ○ 3 patients with 3 <WHO scale ≤4, IL-6 ≤ 63.95 pg/mL, Dyspnea—No (42.9%—Node 14)

The CART algorithm revealed an overall performance of 93.3% with excellent accuracy for moderate (95.9%) and critical forms (98.3%) and a fair performance for critical forms (78.6%).

## 3. Discussion

The mortality rate can vary greatly depending on the geographic location and the availability and quality of healthcare services [29]. Factors such as access to rapid diagnosis and effective therapies play a significant role [29] but also in relation to hthe different age groups and the comorbidities of the patients [29].

As per the Korea Centers for Disease Control and Prevention (KCDC), during the initial wave of SARS-CoV-2 infection in Korea, the mortality rate for the general population was 2.37%. However, for patients aged between 70 and 79 years, the fatality rate was 10.9%, and for those above 80 years, it was 26.6% [30]. During the same period across all European regions, a clear pattern emerges with the cumulative mortality rates (CMRs) escalating as age increases. The highest CMRs were observed in England and Wales, reaching 91.0 (95% CI 89.9–92.1) per 100,000 for men and 72.4 (95% CI 71.4–73.3) per 100,000 for women. Notably high CMRs were also evident in Southern Europe, particularly in Italy (66.2, 95% CI 65.3–67.2 per 100,000 men and 45.4, 95% CI 44.7–46.2 per 100,000 women) and Spain (50.3, 95% CI 49.4–51.2 per 100,000 men and 37.2, 95% CI 36.4–38.0 per 100,000 women). In the majority of regions, the disparity in sex-specific mortality rates grew from under 60 years to the 60–69 years age group but then diminished in older age groups. Although not every change in RRs between consecutive age groups was statistically significant, the differences in the oldest age groups were significant across all regions. The most pronounced gender disparities occurred in the 60–69 years age range in Norway, England and Wales, Germany, and Italy, continuing up to 69 years in Sweden, up to 79 years in the Netherlands, France, and Portugal, spanning 60–79 years in Spain, and peaking at 70–79 years in Denmark. The smallest gender differences were consistently found in the 80+ years age group across all regions [31].

During the onset of the pandemic in China, the general fatality rate was 2.3%. However, for individuals in their 70s, the fatality rate was 8%, and for those above 80 years, it was 14.8% [30]. It is estimated that hospitalized elderly individuals have a fatality rate ranging from 19.2% to 35.9% [32,33]. In New York, among 1.425 patients aged over 60 years, the mortality rate was recorded at 32.7% [34]. In our study, the age group between 70 and 79 years had the highest percentage, accounting for 48.0%—61 patients, of the deceased patients. We noted statistically significant differences between groups related to other cardiovascular diseases, nutritional status, hematological diseases, other neurological/mental or digestive disorders, and other comorbidities. The presence of these factors is associated with an unfavorable outcome. These conditions were also identified in several previous studies, along with cerebrovascular diseases and COPD [35,36,37,38,39].

Regarding other cardiovascular comorbidities, there was a statistically significant favorable outcome in all three age groups, *p* ≤ 0.001. In terms of type 2 diabetes mellitus, there was a statistically significant favorable outcome for the patients in the age group 70–79 years, *p* ≤ 0.028. Patients with diabetes mellitus were closely monitored, and their insulin doses were increased to correct glycemic values. This was particularly important as glucocorticoids were also included in the management during the pro-inflammatory phase of the disease. Other studies have also shown, like in our study, that diabetes mellitus is more commonly found in patients with severe/critical respiratory failure [32]. We have noted that the nutritional status of the patients is a significant factor associated with the need for noninvasive or invasive mechanical ventilation, as seen in other studies in the literature [40,41]. Patients with lower WHO ordinal clinical severity scale categories are associated with lower BMIs. Nutritional status shows significant differences across severity levels (*p* < 0.001).

Our results indicate that with the presence of cough, there was a statistically significant favorable outcome in the age group over 80 years, *p* ≤ 0.049. In terms of the presence of dyspnea in all groups of patients, it was associated with an unfavorable outcome, *p* ≤ 0.001. The presence of headaches in patients in the age group >80 years was associated with a favorable outcome, *p* = 0.001.

Our findings are similar to those of Zhang et al. [38] and Chen et al. [42], which suggested that dyspnea and chest pain are more common in the elderly population. It is important to note that the absence of fever should not be used as an indicator to rule out the suspicion of COVID-19 and postpone the testing of patients for SARS-CoV-2 infection. Leung C. conducted a study to compare the clinical and evolutionary characteristics of deceased elderly patients (89 patients) with survivors (65 cases). The study found that in the age group of 60–69 years, more cases with favorable evolution were recorded, while for those above 80 years, the fatality rate was higher. Cough was found in both groups, while fever was less often associated with the cases that resulted in death. The study also found that lower respiratory tract involvement such as dyspnea, chest pain, and myalgias were more frequently observed in the deceased cases [43].

The correlations made between WHO ordinal scales and inflammatory markers are important to be considered, as they can reveal the severity of a case much earlier, from admission, than the marked respiratory impairment during hospitalization. The neutrophil/lymphocyte ratio is a statistically significant indicator of both the severity of a patient’s respiratory impairment and their risk of death, starting from admission [35]. The neutrophil/lymphocyte ratio is also a significant predictor of the outcome (death) in our study based on our logistic regression analysis model. Each unit increase in neutrophil/lymphocyte ratio results in a 0.106 increase in the log odds of death. The odds ratio (OR) is 1.112, with a 95% CI of 1.025 to 1.206. This means that with each unit increase in neutrophil/lymphocyte ratio, the odds of death increase by 11.2%.

According to Tam EMYY et al., a decrease in the number of lymphocytes and increased values of CPK, LDH, PCR, and PCT are significant in the group of elderly patients with an unfavorable evolution, in which a higher rate of acute renal failure is also found (76.5% vs. 15.5%) along with coinfections (41.2% vs. 13.1%) [44].

Also, Covino et al. have noted the association of the decrease in oxygen saturation with the increase in CRP and LDH levels in the unfavorable evolution related to the advanced age of the patient [45]. Also in our study, LDH is a significant predictor of the outcome (death) based on our logistic regression analysis model. Each unit increase in LDH results in a 0.007 increase in the log odds of death. The odds ratio (OR) is 1.007, with a 95% confidence interval (CI) of 1.002 to 1.012. This means that with each unit increase in LDH, the odds of death increase by 0.7%.

In our study, the laboratory test results that evaluated the degree of inflammation between the different WHO categories according to the outcome group based on the mean values of the biomarkers highlight that except IL-6 all the other biomarkers increase progressively with the severity of the disease and are more increased for the patients with an unfavorable outcome. The differences among severity categories and the outcome group are highly significant (*p*-values < 0.001).

The vulnerability of elderly patients to SARS-CoV-2 infection is due to various factors such as immunosenescence, a decrease in the production of T and B lymphocytes, dysfunction of innate immunity, and a delayed reaction in viral clearance. These factors can act as a persistent trigger of an inadequate immune response, which can favor the onset of a cytokine storm [46]. In addition to the factors mentioned earlier, the subclinical substrate of chronic immune activation (inflammaging) can also contribute to the unfavorable evolution of the infection in elderly patients [47].

From the imaging studies performed in our study, we noted that pneumonia cases initially perceived as mild can develop into more serious forms during a patient’s hospital stay. As a result, it may become necessary to conduct dynamic imaging studies, especially in cases where respiratory function deteriorates.

IL-6 values, along with IL-8 and IL-10, are highly predictive both for SARS-CoV-2 infection severity (86.4%, 95% CI: 72.4–94.8), with a specificity of 94.7% [48], and for the need of case monitoring in intensive care.

The CART algorithm revealed a specific cut-off point for the WHO ordinal scale of 4 to stand out as an important reference value for patients at a high risk of developing critical forms of COVID-19. A WHO ordinal scale of 3 in combination with IL-6 < 63.95 pg/mL and the presence of dyspnea or a WHO ordinal scale of ≤3 in combination with PCT < 0.685 ng/mL and WBCs > 25,880 µL stand out as important factors in developing severe forms of the disease. A procalcitonin (>1.11 ng/mL) in combination with WHO ordinal scale ≤3 or WHO ordinal scale ≤3 in combination with PCT < 0.685 pg/mL and WBCs < 25,880.5 µL stand out as important factors in developing moderate forms of disease. An important observation to be highlighted is the absence of C-reactive protein, LDH, ferritin, or D-dimer and the presence of symptoms in the CART. Inflammation is crucial for multiple organ dysfunctions with exacerbated cytokine production, especially in the lungs [11]. Our results demonstrate elevated inflammatory injury biomarkers and reinforce this hypothesis. The study of Tang et al. [49] presents data on IL-6 and PCT levels across different patient groups, highlighting a clear trend with SARS-CoV-2 severity. In the severe group of patients, IL-6 averages 69.22 pg/mL and PCT averages 0.38 ng/mL. For the critical group, the records show the highest levels: IL-6 averages 160.34 pg/mL, and PCT is at 0.73 ng/mL. Sayah et al. [50] repost optimal cut-off values of the immune inflammatory markers according to the severity of the infection. For IL-6, the optimal point is 44 pg/mL, and for PCT, it is 0.138 ng/mL, lower than the one from our study.

Our study has some limitations that deserve comment. Being a retrospective study, it may not provide a complete understanding of the situation. Also, since it is not a multicenter study, it may introduce some heterogeneity when it comes to including the data. The patient sample is not very large, and it does not come from a wide range of hospitals of all types nationwide, but rather from our county and the surrounding counties, and also that we only analyzed the patients from a specific wave of the COVID-19 pandemic. It is important to acknowledge certain limitations associated with the CART model, given that this study was retrospective, drawing on data from 287 patient records. There is a potential for residual confounding in our methodology. While the results demonstrate notable model performance, the applicability to broader populations warrants additional investigation. Incorporating a larger patient sample would enhance the precision of the model. However, the study has some obvious strengths. The database used in this study is extensive and includes a wide range of clinical and analytical variables at the time of hospital admission. This allowed us to gain a better understanding of this specific group of SARS-CoV-2 patients.

## 4. Materials and Methods

A single-center longitudinal observational cohort study on SARS-CoV-2 infected patients is ongoing at Sibiu Clinical County Hospital, Romania, a county hospital with 1054 beds, dedicated to the treatment of COVID-19 patients since the beginning of this pandemic. In these analyses, we retrospectively included data of all consecutive patients admitted to our hospital between 23 December 2021 and 30 April 2022 (the fourth wave of SARS-CoV-2 infection) with an age over 60 years, all confirmed with SARS-CoV-2 infection (by real-time reverse transcriptase–polymerase chain reaction (RT-PCR) from nasal and pharyngeal swabs). The medical records of the enrolled patients include information such as their demographic characteristics, risk factors and comorbidities, laboratory investigations, treatments, outcomes at the time of discharge from the hospital (alive or death/deceased), and complications. Two independent reviewers assessed data retrieval and consistency.

The primary objectives of our study were to assess the risk factors for an unfavorable outcome. The secondary objective was to assess the clinical and baseline characteristics of the enrolled patients.

All patients received the standard of care according to the local and national guidelines.

The statistical analysis was performed using the IBM SPSS Statistics version 29 software. The analysis of continuous and categorical variables expressed as counts and percentages was performed using univariate descriptive statistics. We performed the Mann–Whitney test to compare continuous variables as the distributions were skewed. In contrast, we used the chi-square test, Fisher’s exact test, or Cramer’s V for categorical variables. To assess the normal distribution of the data, we utilized both Kolmogorov–Smirnov and Shapiro–Wilk tests. A value of *p* = 0.05 or less was considered significant. Categorical (ordinal) variables include COVID-19 clinical forms (infection severity) categorized as mild, moderate, severe, or critical according to clinical symptoms and status on discharge. Dichotomous variables include gender, intensive care unit admission, respiratory support, clinical manifestations (cough, dyspnea, fever, lethargy, chest pain/discomfort, headache, vomiting/nausea, myalgia, arthralgias, diarrhea, fatigue, sore throat, nasal congestion/rhinorrhea, ageusia, anosmia, acute neurological disorders), treatment with remdesivir or favipiravir, immunomodulatory therapy (tocilizumab, anakinra, and corticotherapy), antibiotic therapy (cephalosporins, carbapenems, macrolides, aminoglycosides, polypeptides, oxazolidinones, tetracyclines, fluoroquinolones, and glycopeptides), and antimycotics. Continuous variables include the number of hospitalization days and the results of laboratory performed tests (leukocytes, NLR (neutrophil-to-lymphocyte ratio), ESR, C-reactive protein (CRP), procalcitonin, D-dimer, Ferritin, LDH, fibrinogen). The neutrophil-to-lymphocyte ratio is expressed as the number of neutrophils by the number of lymphocytes ratio, based on a complete blood count (CBC).

The WHO ordinal clinical severity scale was collected at the time of hospital admission and was used to assess the severity of the patients at the time of the initial assessment. The 9 points of the scale are as follows: (0) no clinical or virological evidence of infection; (1) ambulatory, no activity limitation; (2) ambulatory, activity limitation; (3) hospitalized, no oxygen therapy; (4) hospitalized, oxygen mask or nasal prongs; (5) hospitalized, noninvasive mechanical ventilation (NIMV) or high-flow nasal cannula (HFNC); (6) hospitalized, intubation and invasive mechanical ventilation (IMV); (7) hospitalized, IMV + additional support such as pressors or extracardiac membranous oxygenation (ECMO); (8) death [51]. Also, we used the Combined Ordinal Scale of Severity proposed by Rubio-Rivas M. et al. [51], a scale that combines the WHO ordinal scale and the degrees of inflammation, resulting in 6 categories for the hospitalized population with COVID-19: (3a) hospitalized, no oxygen therapy and not high risk of inflammation; (3b) hospitalized, no oxygen therapy and high risk of inflammation; (4a) hospitalized, oxygen mask or nasal cannula and not high risk of inflammation; (4b) hospitalized, oxygen mask or nasal prongs and high risk of inflammation; (5a) hospitalized, NIMV, HFNC, IMV, ICU, ECMO, or pressors and not high risk of inflammation; (5b) hospitalized, NIMV, HFNC, IMV, ICU, ECMO, or pressors and high risk of inflammation [51].

The CT scan severity score used to evaluate the extent of pulmonary involvement was based on the thin-section CT score reported by Chang et al. related to the involved area. There was a score of 0–5 for each lobe (0—None; 1—<5% of a lobe, minimal but not normal; 2—5–25% of lobe; 3—26–49% of lobe; 4—50–75% of lobe; 5—>75% of the lobe), with a total possible score of 0–25 [52].

Reference levels for the used laboratory tests: Urea (mg/dL): 18–55 mg/dL; creatinine (mg/dL): 0.7–1.3 mg/dL; alanine transaminase (U/L): 3–43 U/L; aspartate transferase (U/L): 9–39 U/L; sodium (mEq/L): 136–145 mEq/L; potassium (mEq/L): 3.5–5.1 mEq/L; ferritin (ng/mL): 30–400 ng/mL; D-dimers (ng/mL): 45–499 ng/mL; creatine phosphokinase (IU/L): 10–120 IU/L; lactate dehydrogenase (U/L): 125–220 U.L; IL-6 (pg/mL): 0–7 pg/mL; C-reactive protein (mg/dL): 0.5 mg/dL; fibrinogen (mg/dL): 170–420 mg/dL; erythrocyte sedimentation rate (mm/h): 0–15 mm/h; WBCs (µL): 4000–10,000 µL.

In this research, high-volume clinical chemistry analyzers were used for the accurate detection and measurement of various biomarkers, adapting the detection methods to suit each biomarker’s unique characteristics. For example, urea and creatine phosphokinase were quantified using precise spectrophotometric methods. For creatinine, a colorimetric enzymatic assay proved effective. The enzymatic activities of alanine transaminase and aspartate transaminase were detected either through spectrophotometric analysis or via chemiluminescence techniques. Essential electrolytes like sodium and potassium were accurately measured using sophisticated direct-potentiometric methods. For the sensitive detection of Ferritin and IL-6, the advanced electrochemiluminescence method was employed. Both D-dimers and C-reactive protein levels were quantified with the help of latex-enhanced immunoturbidimetric assays. Fibrinogen concentrations were determined using the reliable coagulometric Clauss assay. Erythrocyte sedimentation rates were captured through cutting-edge automated ESR methods. Finally, white blood cells (WBCs) were counted utilizing a high-tech automated analyzer that integrates flow cytometry with fluorescence, leveraging the latest in laser semiconductor technology and hydrodynamic focusing.

In this research, we leveraged the binary logistic regression and then Classification and Regression Trees (CART) algorithm as a robust machine learning methodology for categorizing and analyzing data, particularly for identifying concealed patterns within the dataset, as substantiated by prior studies [11,53]. A multivariable model based on the binary logistic regression given the main outcome for analysis death(yes/no) was employed first. We have chosen the covariates for our final logistic regression model by carefully considering a balance of statistical significance and the precision of the coefficient estimates, as reflected by their 95% confidence intervals. Covariates with narrower confidence intervals were preferred as they indicate more precise estimates of the effect sizes. Then, CART decision-tree was implemented to supplement our study by examining the risk factors linked to each clinical form. This was particularly important for understanding the variables that might contribute to severe or critical conditions leading to unfavorable outcomes.

In this research, we leveraged the Classification and Regression Trees (CART) algorithm as a robust machine learning methodology for categorizing and analyzing data, particularly for identifying concealed patterns within the dataset, as substantiated by prior studies [11,53]. This algorithm creates a decision tree, which is a hierarchical model of decision-making related to the target variable—here, the overall outcome of our cohort study. The tree is developed in a hierarchical manner, starting from the root and branching out, effectively linking all predictors to forecast the outcome. Each branch bifurcates based on a condition set on the predictor variable, culminating in leaves that symbolize the final decision or outcome.

CART operates as a binary decision tree, utilizing the Gini index and the entropy principle for data segmentation based on predictive variables and purity at each node, progressing from parent to child nodes. The algorithm seeks the most effective division that maximizes node purity through a series of plausible partitioning paths, halting either upon reaching preset termination criteria or when no further improvement in purity is feasible. Its primary focus is to identify the optimal partition point for a predictive variable, enhancing the partitioning criteria in alignment with either the Gini index, in the case of categorical variables, or the least squares deviation (LSD) for continuous variables. The algorithm repetitively finds the best node division, prioritizing the predictive factor that optimizes the partitioning criterion and reduces node impurity significantly. This process is iterated for each child node until no further refinement is possible or specific cessation conditions are met. The minimum threshold for node advancement is typically user-defined, commonly set at 0.0001.

The CART decision tree’s strengths include its flexibility in accommodating various data types, resilience against outliers, and efficient handling of missing data through alternative divisions, making it a highly automated and adaptable method. It is particularly advantageous due to its nonparametric nature, allowing for application to diverse datasets, and its robustness to data anomalies, as it bases partitioning on sample proportions within specified ranges rather than absolute values [34,54,55,56]. Moreover, by employing surrogate splits, CART effectively manages missing data, enabling the construction of robust models even with up to 23% missing data in the target variable for our study group.

For model training, variables with over 23% missing data were excluded, and those with missing values were treated as missing not at random (MNAR) [29,34,54,55,56]. The algorithm was applied using variables that showed statistical significance in univariate analysis.

The predictive model’s accuracy was evaluated using a 10-fold cross-validation technique. The dataset was repeatedly segmented according to the parameter “k = 10”, followed by the computation of the area under the receiver operating characteristic (ROC) curve (AUC) to assess the model’s accuracy.

Written informed consent was obtained from all subjects involved in the study.

Our study was conducted in accordance with the principles of the Declaration of Helsinki and was approved by the Institutional Ethics Committee number (22549/18 September 2020); they also encouraged publishing the article.

## 5. Conclusions

Our study focuses on the relationship between various health factors and clinical outcomes in the elderly population of patients with COVID-19, also including the use of the WHO ordinal clinical severity scale. Patients of all age groups and those with a BMI greater than 30 kg/m^2^ exhibited significantly worse outcomes. A high BMI was correlated with a greater severity of the disease. Dyspnea and fatigue were identified as significant factors associated with worse clinical outcomes, suggesting their prognostic value in mortality. A cross-tabulation analysis showed significant differences in several laboratory parameters between patients with favorable and unfavorable outcomes. Higher mean values of specific biomarkers were observed in patients with worse outcomes. As severity increased, so did the mean values of various biomarkers, notably D-dimers, ferritin, lactate dehydrogenase, and C-reactive protein. IL-6 showed less variation and was not significantly different across severity categories. Our study explored the correlation between comorbidities and severity levels. We conclude that there is a slight but not significant increase in age with severity; significant gender-based differences in clinical outcomes; no significant correlation between hypertension or type 2 diabetes and severity; significant correlations with other cardiovascular diseases and chronic kidney disease; hematological malignancies, but not solid tumors, correlated significantly with higher severity; no significant correlations with stroke, neurological disorders, acute psychiatric pathology, or other mental disorders. Our study used the CART algorithm to identify critical reference points on the WHO ordinal scale for predicting the risk of severe COVID-19. Specific combinations of WHO scale values, IL-6 levels, dyspnea, procalcitonin (PCT), and white blood cell counts (WBCs) were identified as significant in predicting the development of severe or moderate forms of the disease. In summary, the study provides a comprehensive analysis of how various health factors, symptoms, and biomarkers correlate with the severity of COVID-19, offering insights into potential prognostic indicators and aiding in a better understanding of the disease progression.

## Figures and Tables

**Table 1:** Descriptive statistics of the enrolled patients.

Variables	Statistical Outcome				
	Mean	Median	Std. dev.	Min.	Max.
**Age (years)**	74.58	74.00	7.940	60	94
**Length of hospitalization (days)**	10.49	9	6.528	1	54
	No. of patients (*n* = %)				
**Outcome**					
**Deceased**	127 (44.3%)				
**Alive**	160 (55.7%)				
**Gender**					
**Female**	160 (55.7%)				
**Male**	127 (44.3%)				
**Age group (years)**					
**60–69**	81 (28.2%)				
**70–79**	123 (42.9%)				
**>80**	83 (28.9%)				
**Form of COVID-19 pneumonia based on the severity grade classification**					
**Mild**	17 (5.9%)				
**Moderate**	97 (33.8%)				
**Sever**	56 (19.5%)				
**Critical**	117 (40.8%)				
**Intensive care unit hospitalization**					
**No**	166 (57.8%)				
**Yes**	121 (42.2%)				
**Vaccination status**					
**Vaccinated**	233 (81.2%)				
**Not vaccinated**	54 (18.8%)				
**Required oxygen therapy by type**					
**No**	116 (40.4%)				
**Continuous positive airway pressure**	28 (9.8%)				
**High-flow nasal cannula oxygen**	15 (5.2%)				
**Oxygen mask**	49 (17.1%)				
**Invasive mechanical ventilation**	79 (27.5%)				

**Table 2:** Cross-tabulation between clinical manifestations and outcome.

		Outcome		
		Deceased	Alive	Fisher’s Exact Test (Exact Sig. (2-Sided))
**Fever**	NO	81	100	0.902
	YES	46	60	
**Cough**	NO	58	54	0.051
	YES	69	106	
**Dyspnea**	NO	15	98	<0.001
	YES	112	62	
**Headache**	NO	104	118	0.119
	YES	23	42	
**Lethargy**	NO	92	108	0.438
	YES	35	52	
**Fatigue**	NO	69	54	<0.001
	YES	58	196	
**Nausea**	NO	114	150	0.275
	YES	13	10	
**Vomiting**	NO	121	114	0.119
	YES	6	16	
**Diarrhea**	NO	121	144	0.119
	YES	6	16	
**Abdominal pain**	NO	123	146	0.084
	YES	4	14	
**Sore throat**	NO	123	138	0.002
	YES	4	22	
**Anosmia**	NO	113	154	0.020
	YES	14	6	
**Ageusia**	NO	117	158	0.007
	YES	10	2	
**Viral exanthema**	NO	127	160	-
**Myalgia**	NO	112	132	0.188
	YES	15	28	
**Arthralgias**	NO	123	154	1
	YES	4	6	
**Diaphoresis**	NO	121	144	0.119
	YES	6	16	
**Nasal congestion/rhinorrhea**	NO	124	144	0.015
	YES	3	16	
**Epistaxis**	NO	126	160	0.443
	YES	1	0	
**Chest pain/discomfort**	NO	122	146	0.150
	YES	5	14	
**Acute neurological disorders**	NO	104	142	0.126
	YES	23	18	
**Acute cardiac disorders**	NO	114	142	0.850
	YES	13	18	

**Table 3:** Laboratory parameters and imaging diagnostic scores within the studied population.

	Minimum	Maximum	Mean	Std. Deviation
**Urea (mg/dL)**	12.00	427.00	67.19	56.20
**Creatinine (mg/dL)**	0.57	13.22	1.94	2.09
**Alanine transaminase (U/L)**	8.00	8004.00	82.80	494.97
**Aspartate transferase (U/L)**	6.00	5887.00	63.95	366.53
**Sodium (mEq/L)**	121	154	138.61	4.42
**Potassium (mEq/L)**	2.39	8.12	4.28	0.92
**Ferritin (ng/mL)**	9.00	3375.40	569.71	604.27
**D-Dimers (ng/mL)**	133.60	50,000.00	4417.15	9430.71
**Creatine phosphokinase (IU/L)**	13	9468	370.98	963.71
**Lactate dehydrogenase (U/L)**	108	7199	464.25	557.98
**IL-6 (pg/mL)**	4.04	710.10	87.98	132.06
**C-reactive protein (mg/dL)**	0.47	557.00	96.71	96.64
**Fibrinogen (mg/dL)**	81	1274	497.38	199.35
**Erythrocyte sedimentation rate (mm/h)**	1	120	38.82	28.20
**WBCs (µL)**	13	66,700	16,289.66	15,015.44
**Neutrophil-to-lymphocyte ratio**	0.10	90.73	11.96	12.53
**Pulmonary CT-scan score**	0	25	10.40	8.50

**Table 4:** WHO ordinal clinical severity scale.

**Mean**	**WHO ordinal clinical severity scale**	**3**	**4**	**5**	**6**	**7**	**Total**	**Sig.**
	**D-Dimers (ng/mL)**	1776.70	3586.90	9622.56	6478.49	5827.49	4417.15	<0.001
	**Ferritin (ng/mL)**	401.31	451.64	964.39	955.14	1249.89	569.71	<0.001
	**Lactate dehydrogenase (U/L)**	257.94	501.35	687.28	644.20	645.69	464.25	<0.001
	**IL-6 (pg/mL)**	94.04	24.04	153.71	92.20	80.78	87.98	0.105
	**C-reactive protein (mg/dL)**	54.12	77.44	133.62	144.51	159.91	96.71	<0.001
	**Neutrophil-to-lymphocyte ratio**	6.45	10.96	14.18	20.55	18.30	11.96	<0.001

**Table 5:** WHO ordinal clinical severity scale by outcome.

Mean							
WHO Ordinal Clinical Severity Scale	OUTCOME	D-Dimers	Ferritin	LDH	IL-6	C-Reactive Protein	Neutrophil-To-Lymphocyte Ratio
**3**	DEATH	2722.98	1197.35	721.38	165.81	152.65	26.97
	ALIVE	1715.37	386.57	222.96	65.33	46.76	4.94
	Total	1776.70	401.32	257.94	94.04	54.12	6.45
**4**	DEATH	14,279.67	1036.00	1468.75	51.40	160.56	10.77
	ALIVE	1761.31	437.39	312.59	20.13	63.25	11.00
	Total	3586.91	451.64	501.35	24.04	77.44	10.97
**5**	DEATH	12,792.42	759.81	813.07	83.90	147.22	16.99
	ALIVE	1265.69	1113.18	367.09	258.43	94.07	6.00
	Total	9622.57	964.39	687.28	153.71	133.62	14.18
**6**	DEATH	6478.50	955.15	644.20	92.20	144.51	20.56
	Total	6478.50	955.15	644.20	92.20	144.51	20.56
**7**	DEATH	5827.49	1249.89	645.69	80.78	159.92	18.31
	Total	5827.49	1249.89	645.69	80.78	159.92	18.31
**Total**	DEATH	8045.06	1038.65	747.56	91.25	151.11	18.86
	ALIVE	1696.23	449.55	256.25	82.86	54.28	6.51
	Total	4417.15	569.72	464.25	87.98	96.71	11.96
**Sig.**		<0.001	<0.001	<0.001	0.105	<0.001	<0.001

**Table 6:** Cross-tabulation between WHO ordinal clinical severity scale, health factors, and outcome.

WHO Ordinal Clinical Severity Scale		3	4	5	6	7	Sig.
Age (Years, Mean)		73.94	75.29	75.07	74.26	75.47	0.769
**Gender**	**Female**	69	31	18	29	13	0.011
	**Male**	47	18	25	14	23	
**Hypertension**	**No**	25	12	11	13	14	0.314
	**Yes**	91	37	32	39	22	
**Other cardiovascular diseases**	**No**	25	4	17	23	21	<0.001
	**Yes**	91	45	26	29	15	
**Type 2 diabetes mellitus**	**No**	68	29	23	24	22	0.122
	**Yes**	48	20	20	9	14	
**Chronic kidney disease**	**No**	94	36	31	41	24	0.014
	**Yes**	22	13	12	2	12	
**Nutritional status**	**BMI 18.5–24.9 kg/m^2^**	62	17	13	17	13	<0.001
	**BMI 25–29.9 kg/m^2^**	44	27	13	10	5	
	**BMI > 30 kg/m^2^**	10	5	17	16	18	
**Solid tumors**	**No**	99	47	41	38	32	0.204
	**Yes**	17	2	2	5	4	
**Hematological malignancy**	**No**	88	39	43	41	34	<0.001
	**Yes**	28	19	9	2	2	
**Stroke**	**No**	96	45	36	42	31	0.104
	**Yes**	20	4	7	1	5	
**Other neurological disorders**	**No**	90	40	36	37	34	0.202
	**Yes**	26	9	7	6	2	
**Acute psychiatric pathology**	**No**	110	43	42	40	31	0.170
	**Yes**	6	6	1	3	5	
**Other mental disorders**	**No**	104	43	42	42	35	0.108
	**Yes**	12	6	1	1	1	

## Data Availability

The data presented in this study are available upon reasonable request from the corresponding author.

## Appendix A

**Table A1:** Frequency table for comorbidities.

Variables	No. of Patients	
	n	%
**Hypertension**		
**No**	75	26.1
**Yes**	212	73.9
**Other cardiovascular diseases**		
**No**	90	31.4
**Yes**	197	68.6
**Type 2 diabetes mellitus**		
**No**	176	61.3
**Yes**	111	38.7
**Obesity**		
**Normal weight**	122	42.5
**Overweight**	99	34.5
**Yes**	66	23.0
**Chronic kidney disease**		
**No**	226	78.7
**Yes**	61	21.3
**Solid tumors**		
**No**	257	89.5
**Yes**	30	10.5
**Hematological malignancy**		
**No**	245	85.4
**Yes**	42	14.6
**Stroke**		
**No**	250	87.1
**Yes**	37	12.9
**Other neurological disorders**		
**No**	237	82.6
**Yes**	50	17.4
**Metabolic diseases**		
**No**	216	75.3
**Yes**	71	24.7
**Acute psychiatric pathology**		
**No**	266	92.7
**Yes**	21	7.3
**Other mental disorders**		
**No**	266	92.7
**Yes**	21	7.3
**Digestive disorders**		
**No**	194	67.6
**Yes**	93	32.4
**Endocrine disorders**		
**No**	252	87.8
**Yes**	35	12.2
**Urological disorders**		
**No**	251	87.5
**Yes**	36	12.5
**Autoimmune diseases**		
**No**	277	96.5
**Yes**	10	3.5
**Other comorbidities**		
**No**	157	54.7
**Yes**	130	45.3

**Table A2:** Cross-tabulation between comorbidities and outcome.

		Outcome		
		Deceased	Alive	Fisher’s Exact Test (Exact Sig. (2-Sided))
**Hypertension**	NO	37	38	0.344
	YES	90	122	
**Other cardiovascular diseases**	NO	68	22	<0.001
	YES	59	138	
**Type 2 diabetes mellitus**	NO	84	92	0.145
	YES	43	68	
**Chronic kidney disease**	NO	96	130	0.250
	YES	31	30	
**Nutritional status**	BMI 18.5–24.9 kg/m^2^	44	78	<0.001
	BMI 25–29.9 kg/m^2^	25	74	
	BMI > 30 kg/m^2^	58	8	
**Metabolic diseases**	NO	94	122	0.681
	YES	33	38	
**Solid tumors**	NO	113	144	0.847
	YES	14	16	
**Hematological malignancy**	NO	123	122	<0.001
	YES	4	38	
**Stroke**	NO	116	134	0.075
	YES	11	26	
**Other neurological disorders**	NO	113	124	0.012
	YES	14	36	
**Acute psychiatric pathology**	NO	118	148	1
	YES	9	12	
**Other mental disorders**	NO	124	142	0.005
	YES	3	18	
**Digestive disorders**	NO	112	82	<0.001
	YES	15	78	
**Urological disorders**	NO	113	138	0.591
	YES	14	22	
**Endocrine disorders**	NO	106	146	0.068
	YES	21	14	
**Autoimmune diseases**	NO	123	154	1
	YES	4	6	
**Other comorbidities**	NO	91	66	<0.001
	YES	36	94	

**Table A3:** Cross-tabulation between other cardiovascular comorbidities by outcome and age by group.

Chi-Square Tests						
Age by Group (Years)		Value	df	Asymptotic Significance (2-Sided)	Exact Sig. (2-Sided)	Exact Sig. (1-Sided)
60–69	Pearson chi-square	8.784 ^c^	1	0.003		
	Likelihood ratio	8.820	1	0.003		
	Fisher’s exact test				0.005	0.003
	No. of valid cases	81				
70–79	Pearson chi-square	35.143 ^d^	1	<0.001		
	Likelihood ratio	38.014	1	<0.001		
	Fisher’s exact test				<0.001	<0.001
	No. of valid cases	123				
>80	Pearson chi-square	13.095 ^e^	1	<0.001		
	Likelihood ratio	13.696	1	<.001		
	Fisher’s exact test				<0.001	<0.001
	No. of valid cases	83				
Total	Pearson chi-square	52.086 ^a^	1	<0.001		
	Likelihood ratio	53.446	1	<0.001		
	Fisher’s exact test				<0.001	<0.001
	No. of valid cases	287				

^a^ 0 cells (0.0%) have an expected count of less than 5. The minimum expected count is 39.83. ^c^ 0 cells (0.0%) have an expected count of less than 5. The minimum expected count is 12.63. ^d^ 0 cells (0.0%) have an expected count of less than 5. The minimum expected count is 21.33. ^e^ 0 cells (0.0%) have an expected count of less than 5. The minimum expected count is 5.90.

**Table A4:** Cross-tabulation between type 2 diabetes mellitus by outcome and age by group.

Chi-Square Tests						
Age by Group (Years)		Value	df	Asymptotic Significance (2-Sided)	Exact Sig. (2-Sided)	Exact Sig. (1-Sided)
60–69	Pearson chi-square	0.060 ^c^	1	0.806		
	Likelihood ratio	0.060	1	0.806		
	Fisher’s exact test				0.817	0.495
	No. of valid cases	81				
70–79	Pearson chi-square	5.306 ^d^	1	0.021		
	Likelihood ratio	5.352	1	0.021		
	Fisher’s exact test				0.028	0.017
	No. of valid cases	123				
>80	Pearson chi-square	0.091 ^e^	1	0.763		
	Likelihood ratio	0.091	1	0.763		
	Fisher’s exact test				0.820	0.474
	No. of valid cases	83				
Total	Pearson chi-square	2.229 ^a^	1	0.135		
	Likelihood ratio	2.240	1	0.134		
	Fisher’s exact test				0.145	0.085
	No. of valid cases	287				

^a^ 0 cells (0.0%) have an expected count of less than 5. The minimum expected count is 49.12. ^c^ 0 cells (0.0%) have an expected count of less than 5. The minimum expected count is 11.48. ^d^ 0 cells (0.0%) have an expected count of less than 5. The minimum expected count is 25.29. ^e^ 0 cells (0.0%) have an expected count of less than 5. The minimum expected count is 12.65.

**Table A5:** Cross-tabulation between nutritional status by outcome and age by group.

Chi-Square Tests				
Age by Group (Years)		Value	df	Asymptotic Significance (2-Sided)
60–69	Pearson chi-square	19.682 ^b^	2	<0.001
	Likelihood ratio	19.862	2	<0.001
	Linear-by-linear association	11.992	1	<0.001
	No. of valid cases	81		
70–79	Pearson chi-square	38.245 ^c^	2	<0.001
	Likelihood ratio	44.062	2	<0.001
	Linear-by-linear association	21.280	1	<0.001
	No. of valid cases	123		
>80	Pearson chi-square	11.298 ^d^	2	0.004
	Likelihood ratio	11.791	2	0.003
	Linear-by-linear association	3.037	1	0.081
	No. of valid cases	83		
Total	Pearson chi-square	68.721 ^a^	2	<0.001
	Likelihood ratio	73.898	2	<0.001
	Linear-by-linear association	34.308	1	<0.001
	No. of valid cases	287		

^a^ 0 cells (0.0%) have an expected count of less than 5. The minimum expected count is 29.21. ^b^ 0 cells (0.0%) have an expected count of less than 5. The minimum expected count is 7.65. ^c^ 0 cells (0.0%) have an expected count of less than 5. The minimum expected count is 16.86. ^d^ 0 cells (0.0%) have an expected count of less than 5. The minimum expected count is 5.06.

**Table A6:** Cross-tabulation between hematological malignancies by outcome and age by group.

Chi-Square Tests						
Age by Group (Years)		Value	df	Asymptotic Significance (2-Sided)	Exact Sig. (2-Sided)	Exact Sig. (1-Sided)
60–69	Pearson chi-square	9.554 ^c^	1	0.002		
	Likelihood ratio	11.712	1	<0.001		
	Fisher’s exact test				0.002	0.001
	No. of valid cases	81				
70–79	Pearson chi-square	10.123 ^d^	1	0.001		
	Likelihood ratio	11.249	1	<0.001		
	Fisher’s exact test				0.002	0.001
	No. of valid cases	123				
>80	Pearson chi-square	3.993 ^e^	1	0.046		
	Likelihood ratio	4.640	1	0.031		
	Fisher’s exact test				0.072	0.045
	No. of valid cases	83				
Total	Pearson chi-square	24.051 ^a^	1	<0.001		
	Likelihood ratio	28.008	1	<0.001		
	Fisher’s exact test				<0.001	<0.001
	No. of valid cases	287				

^a^ 0 cells (0.0%) have an expected count of less than 5. The minimum expected count is 18.59. ^c^ 0 cells (0.0%) have an expected count of less than 5. The minimum expected count is 6.51. ^d^ 0 cells (0.0%) have an expected count of less than 5. The minimum expected count is 7.93. ^e^ 1 cells (25.0%) have an expected count of less than 5. The minimum expected count is 3.80.

**Table A7:** Cross-tabulation between digestive disorders by outcome and age by group.

Chi-Square Tests						
Age by Group (Years)		Value	df	Asymptotic Significance (2-Sided)	Exact Sig. (2-Sided)	Exact Sig. (1-Sided)
60–69	Pearson chi-square	8.457 ^c^	1	0.004		
	Likelihood ratio	9.042	1	0.003		
	Fisher’s exact test				0.004	0.003
	No. of valid cases	81				
70–79	Pearson chi-square	30.151 ^d^	1	<0.001		
	Likelihood ratio	33.302	1	<0.001		
	Fisher’s exact test				<0.001	<0.001
	No. of valid cases	123				
>80	Pearson chi-square	7.453 ^e^	1	0.006		
	Likelihood ratio	7.839	1	0.005		
	Fisher’s exact test				0.009	0.006
	No. of valid cases	83				
Total	Pearson chi-square	44.105 ^a^	1	<0.001		
	Likelihood ratio	47.605	1	<0.001		
	Fisher’s exact test				<0.001	<0.001
	No. of valid cases	287				

^a^ 0 cells (0.0%) have an expected count of less than 5. The minimum expected count is 41.15. ^c^ 0 cells (0.0%) have an expected count of less than 5. The minimum expected count is 11.10. ^d^ 0 cells (0.0%) have an expected count of less than 5. The minimum expected count is 17.85. ^e^ 0 cells (0.0%) have an expected count of less than 5. The minimum expected count is 11.81.

**Table A8:** Cross-tabulation between other comorbidities by outcome and age by group.

Chi-Square Tests						
Age by Group (Years)		Value	df	Asymptotic Significance (2-Sided)	Exact Sig. (2-Sided)	Exact Sig. (1-Sided)
60–69	Pearson chi-square	7.065 ^c^	1	0.008		
	Likelihood ratio	7.291	1	0.007		
	Fisher’s exact test				0.011	0.007
	No. of valid cases	81				
70–79	Pearson chi-square	16.888 ^d^	1	<0.001		
	Likelihood ratio	17.345	1	<0.001		
	Fisher’s exact test				<0.001	<0.001
	No. of valid cases	123				
>80	Pearson chi-square	3.636 ^e^	1	0.057		
	Likelihood ratio	3.668	1	0.055		
	Fisher’s exact test				0.076	0.046
	No. of valid cases	83				
Total	Pearson chi-square	26.413 ^a^	1	<0.001		
	Likelihood ratio	27.007	1	<0.001		
	Fisher’s exact test				<0.001	<0.001
	No. of valid cases	287				

^a^ 0 cells (0.0%) have an expected count of less than 5. The minimum expected count is 57.53. ^c^ 0 cells (0.0%) have an expected count of less than 5. The minimum expected count is 13.78. ^d^ 0 cells (0.0%) have an expected count of less than 5. The minimum expected count is 26.28. ^e^ 0 cells (0.0%) have an expected count of less than 5. The minimum expected count is 17.29.

**Table A9:** Clinical manifestations at the time of admission into the hospital.

**Fever**		
	Frequency	Percent
NO	181	63.1
YES	106	36.9
**Cough**		
NO	112	39.0
YES	175	61.0
**Dyspnea**		
NO	113	39.4
YES	174	60.6
**Headache**		
NO	222	77.4
YES	65	22.6
**Lethargy**		
NO	200	69.7
YES	87	30.3
**Fatigue**		
NO	123	42.9
YES	164	57.1
**Nausea**		
NO	264	92.0
YES	23	8.0
**Vomiting**		
NO	265	92.3
YES	22	7.7
**Diarrhea**		
NO	265	92.3
YES	22	7.7
**Abdominal pain**		
NO	269	93.7
YES	18	6.3
**Sore throat**		
NO	261	90.9
YES	26	9.1
**Anosmia**		
NO	267	93.0
YES	20	7.0
**Ageusia**		
NO	275	95.8
YES	12	4.2
**Viral exanthema**		
NO	287	100.0
**Myalgia**		
NO	244	85.0
YES	43	15.0
**Arthralgias**		
NO	277	96.5
YES	10	3.5
Diaphoresis		
NO	265	92.3
YES	22	7.7
**Nasal congestion/rhinorrhea**		
NO	268	93.4
YES	19	6.6
**Epistaxis**		
NO	286	99.7
YES	1	0.3
**Chest pain/discomfort**		
NO	268	93.4
YES	19	6.6
**Acute neurological disorders**		
NO	246	85.7
YES	41	14.3
**Acute cardiac disorders**		
NO	256	89.2
YES	31	10.8

**Table A10:** Cross-tabulation between cough by outcome and age by group.

Chi-Square Tests						
Age by Group (Years)		Value	df	Asymptotic Significance (2-Sided)	Exact Sig. (2-Sided)	Exact Sig. (1-Sided)
60–69	Pearson chi-square	1.421 ^c^	1	0.233		
	Continuity correction ^b^	0.913	1	0.339		
	Likelihood ratio	1.411	1	0.235		
	Fisher’s exact test				0.248	0.170
	Linear-by-linear association	1.404	1	0.236		
	No. of valid cases	81				
70–79	Pearson chi-square	0.401 ^d^	1	0.526		
	Continuity correction ^b^	0.197	1	0.657		
	Likelihood ratio	0.401	1	0.526		
	Fisher’s exact test				0.574	0.328
	Linear-by-linear association	0.398	1	0.528		
	No. of valid cases	123				
>80	Pearson chi-square	4.114 ^e^	1	0.043		
	Continuity correction ^b^	3.260	1	0.071		
	Likelihood ratio	4.140	1	0.042		
	Fisher’s exact test				0.049	0.035
	Linear-by-linear association	4.064	1	0.044		
	No. of valid cases	83				
Total	Pearson chi-square	4.227 ^a^	1	0.040		
	Continuity correction ^b^	3.741	1	0.053		
	Likelihood ratio	4.222	1	0.040		
	Fisher’s exact test				0.051	0.027
	Linear-by-linear association	4.212	1	0.040		
	No. of valid cases	287				

^a^ 0 cells (0.0%) have an expected count of less than 5. The minimum expected count is 49.56. ^b^ Computed only for a 2 × 2 table. ^c^ 0 cells (0.0%) have an expected count of less than 5. The minimum expected count is 11.48. ^d^ 0 cells (0.0%) have an expected count of less than 5. The minimum expected count is 21.33. ^e^ 0 cells (0.0%) have an expected count of less than 5. The minimum expected count is 16.45.

**Table A11:** Cross-tabulation between dyspnea by outcome and age by group.

Chi-Square Tests						
Age by Group (Years)		Value	df	Asymptotic Significance (2-Sided)	Exact Sig. (2-Sided)	Exact Sig. (1-Sided)
60–69	Pearson chi-square	29.359 ^c^	1	<0.001		
	Continuity correction ^b^	26.919	1	<0.001		
	Likelihood ratio	33.769	1	<0.001		
	Fisher’s exact test				<0.001	<0.001
	Linear-by-linear association	28.996	1	<0.001		
	No. of valid cases	81				
70–79	Pearson chi-square	42.745 ^d^	1	<0.001		
	Continuity correction ^b^	40.172	1	<0.001		
	Likelihood ratio	51.338	1	<0.001		
	Fisher’s exact test				<0.001	<0.001
	Linear-by-linear association	42.398	1	<0.001		
	No. of valid cases	123				
>80	Pearson chi-square	6.446 ^e^	1	0.011		
	Continuity correction ^b^	5.367	1	0.021		
	Likelihood ratio	6.536	1	0.011		
	Fisher’s exact test				0.015	0.010
	Linear-by-linear association	6.368	1	0.012		
	No. of valid cases	83				
Total	Pearson chi-square	72.496 ^a^	1	<0.001		
	Continuity correction ^b^	70.440	1	<0.001		
	Likelihood ratio	78.926	1	<0.001		
	Fisher’s exact test				<0.001	<0.001
	Linear-by-linear association	72.244	1	<0.001		
	No. of valid cases	287				

^a^ 0 cells (0.0%) have an expected count of less than 5. The minimum expected count is 50.00. ^b^ Computed only for a 2 × 2 table. ^c^ 0 cells (0.0%) have an expected count of less than 5. The minimum expected count is 13.78. ^d^ 0 cells (0.0%) have an expected count of less than 5. The minimum expected count is 17.36. ^e^ 0 cells (0.0%) have an expected count of less than 5. The minimum expected count is 17.29.

**Table A12:** Cross-tabulation between headache by outcome and age by group.

Chi-Square Tests						
Age by Group (Years)		Value	df	Asymptotic Significance (2-Sided)	Exact Sig. (2-Sided)	Exact Sig. (1-Sided)
60–69	Pearson chi-square	29.359 ^c^	1	<0.001		
	Continuity correction ^b^	26.919	1	<0.001		
	Likelihood ratio	33.769	1	<0.001		
	Fisher’s exact test				<0.001	<0.001
	Linear-by-linear association	28.996	1	<0.001		
	No. of valid cases	81				
70–79	Pearson chi-square	42.745 ^d^	1	<0.001		
	Continuity correction ^b^	40.172	1	<0.001		
	Likelihood ratio	51.338	1	<0.001		
	Fisher’s exact test				<0.001	<0.001
	Linear-by-linear association	42.398	1	<0.001		
	No. of valid cases	123				
>80	Pearson chi-square	6.446 ^e^	1	0.011		
	Continuity correction ^b^	5.367	1	0.021		
	Likelihood ratio	6.536	1	0.011		
	Fisher’s exact test				0.015	0.010
	Linear-by-linear association	6.368	1	0.012		
	No. of valid cases	83				
Total	Pearson chi-square	72.496 ^a^	1	<0.001		
	Continuity correction ^b^	70.440	1	<0.001		
	Likelihood ratio	78.926	1	<0.001		
	Fisher’s exact test				<0.001	<0.001
	Linear-by-linear association	72.244	1	<0.001		
	No. of valid cases	287				

^a^ 0 cells (0.0%) have an expected count of less than 5. The minimum expected count is 50.00. ^b^ Computed only for a 2 × 2 table. ^c^ 0 cells (0.0%) have an expected count of less than 5. The minimum expected count is 13.78. ^d^ 0 cells (0.0%) have an expected count of less than 5. The minimum expected count is 17.36. ^e^ 0 cells (0.0%) have an expected count of less than 5. The minimum expected count is 17.29.

**Table A13:** Cross-tabulation between other fatigue by outcome and age by group.

Chi-Square Tests						
Age by Group (Years)		Value	df	Asymptotic Significance (2-Sided)	Exact Sig. (2-Sided)	Exact Sig. (1-Sided)
60–69	Pearson chi-square	3.474 ^c^	1	0.062		
	Continuity correction ^b^	2.674	1	0.102		
	Likelihood ratio	3.497	1	0.061		
	Fisher’s exact test				0.072	0.051
	Linear-by-linear association	3.431	1	0.064		
	No. of valid cases	81				
70–79	Pearson chi-square	2.344 ^d^	1	0.126		
	Continuity correction ^b^	1.822	1	0.177		
	Likelihood ratio	2.351	1	0.125		
	Fisher’s exact test				0.149	0.088
	Linear-by-linear association	2.325	1	0.127		
	No. of valid cases	123				
>80	Pearson chi-square	8.476 ^e^	1	0.004		
	Continuity correction ^b^	7.162	1	0.007		
	Likelihood ratio	8.499	1	0.004		
	Fisher’s exact test				0.005	0.004
	Linear-by-linear association	8.374	1	0.004		
	No. of valid cases	83				
Total	Pearson chi-square	12.246 ^a^	1	<0.001		
	Continuity correction ^b^	11.420	1	<0.001		
	Likelihood ratio	12.288	1	<0.001		
	Fisher’s exact test				<0.001	<0.001
	Linear-by-linear association	12.203	1	<0.001		
	No. of valid cases	287				

^a^ 0 cells (0.0%) have an expected count of less than 5. The minimum expected count is 54.43. ^b^ Computed only for a 2 × 2 table. ^c^ 0 cells (0.0%) have an expected count of less than 5. The minimum expected count is 14.93. ^d^ 0 cells (0.0%) have an expected count of less than 5. The minimum expected count is 27.77. ^e^ 0 cells (0.0%) have an expected count of less than 5. The minimum expected count is 11.81.

**Table A14:** Cross-tabulation between sore throat by outcome and age by group.

Chi-Square Tests						
Age by Group (Years)		Value	df	Asymptotic Significance (2-Sided)	Exact Sig. (2-Sided)	Exact Sig. (1-Sided)
60–69	Pearson chi-square	6.129 ^c^	1	0.013		
	Continuity correction ^b^	4.684	1	0.030		
	Likelihood ratio	7.416	1	0.006		
	Fisher’s exact test				0.014	0.011
	Linear-by-linear association	6.054	1	0.014		
	No. of valid cases	81				
70–79	Pearson chi-square	0.667 ^d^	1	0.414		
	Continuity correction ^b^	0.159	1	0.690		
	Likelihood ratio	0.680	1	0.410		
	Fisher’s exact test				0.680	0.348
	Linear-by-linear association	0.662	1	0.416		
	No. of valid cases	123				
>80	Pearson chi-square	2.437 ^e^	1	0.118		
	Continuity correction ^b^	1.348	1	0.246		
	Likelihood ratio	2.762	1	0.097		
	Fisher’s exact test				0.230	0.121
	Linear-by-linear association	2.408	1	0.121		
	No. of valid cases	83				
Total	Pearson chi-square	9.657 ^a^	1	0.002		
	Continuity correction ^b^	8.413	1	0.004		
	Likelihood ratio	10.779	1	0.001		
	Fisher’s exact test				0.002	0.001
	Linear-by-linear association	9.623	1	0.002		
	No. of valid cases	287				

^a^ 0 cells (0.0%) have an expected count of less than 5. The minimum expected count is 11.51. ^b^ Computed only for a 2 × 2 table. ^c^ 1 cells (25.0%) have an expected count of less than 5. The minimum expected count is 4.98. ^d^ 2 cells (50.0%) have an expected count of less than 5. The minimum expected count is 2.98. ^e^ 2 cells (50.0%) have an expected count of less than 5. The minimum expected count is 2.95.

**Table A15:** Cross-tabulation between laboratory parameters and imaging diagnostic scores within the studied population and outcome.

Parameter	Outcome						Sig.
	Death			Alive			
	Mean	95% CI	Std. Deviation	Mean	95% CI	Std. Deviation	
**Urea (mg/dL)**	76.11	51.55–81.92	55.587	60.11	40.34–74.65	55.85	0.000
**Creatinine (** **mg/dL)**	2.37	1.35–2.87	2.096	1.60	0.84–1.77	2.029	0.016
**Alanine transaminase (U/L)**	139.47	38.97–61.46	740.615	37.82	35.25–59.45	37.768	0.002
**Aspartate transferase (U/L)**	106.05	27.93–47.11	548.477	30.53	26.87–46.41	26.98	0.084
**Sodium (mEq/L)**	139.00	135.85–140.23	5.519	138.30	135.80–138.06	3.29	0.083
**Potassium (mEq/L)**	4.29	4.03–4.72	0.847	4.28	4.04–4.22	0.99	0.184
**Ferritin (ng/mL)**	1038.65	645.99–1312.12	763.608	449.55	362.84–885.58	491.30	0.882
**D-Dimers (ng/mL)**	8045.05	181.73–11941.54	13,489.20	1696.22	1387.12–2798.32	1579.68	0.000
**Creatine phosphokinase (IU/L)**	528.13	156.15–387.06	1173.80	254.10	153.85–238.58	754.37	0.019
**Lactate dehydrogenase (U/L)**	747.56	393.03–626.53	764.03	256.25	276.51–356.64	104.66	0.000
**IL-6 (pg/mL)**	91.25	45.42–85.62	97.89	82.85	14.93–155.06	174.52	0.788
**C-reactive protein (mg/dL)**	151.11	134.34–167.88	94.35	54.28	42.55–66.01	74.86	0.000
**Fibrinogen (mg/dL)**	541.91	502.07–538.49	228.16	465.10	466.48–573.16	169.07	0.001
**Erythrocyte sedimentation rate (mm/h)**	52.38	47.58–71.29	31.25	34.96	35.75–56.25	26.12	0.000
**WBCs (µL)**	24330.63	24,171.52–37,781.97	17143.76	9928.13	6115.34–9789.23	8979.72	0.000
**Neutrophil-to-lymphocyte ratio**	18.85	10.39–19.41	14.07	6.5068	5.51–14.34	7.620	0.000
**Pulmonary CT-scan score**	15.91	15.18–19.78	7.96	6.04	7.39–12.75	6.02	0.000

**Table A16:** Cross-tabulation between laboratory parameters and imaging diagnostic scores within the studied population by outcome and age by group.

**Mean**	**Outcome**	**Death**			**Alive**			**Total**			**Sig.**
	**Age by Group (Years)**	**60–69**	**70–79**	**>80**	**60–69**	**70–79**	**>80**	**60–69**	**70–79**	**>80**	
	**D-Dimers (ng/mL)**	9556.07	8172.11	6543.25	1737.29	1377.87	2064.67	4607.47	4632.25	3921.64	0.000
	**Urea (mg/dL)**	59.87	80.05	83.63	68.48	50.94	63.25	65.19	65.37	71.84	0.016
	**Creatinine (mg/dL)**	2.87	2.05	2.50	2.02	1.39	1.45	2.35	1.72	1.89	0.002
	**Alanine transaminase (U/L)**	312.23	63.61	118.69	28.76	45.84	36.92	137.25	54.65	71.40	0.084
	**Aspartate transferase (U/L)**	231.32	50.80	91.40	24.44	32.10	34.88	103.62	41.37	58.71	0.083
	**Sodium (mEq/L)**	139.71	138.26	139.66	137.36	138.71	138.75	138.26	138.49	139.13	0.184
	**Potassium (mEq/L)**	4.23	4.34	4.29	4.44	4.23	4.19	4.36	4.28	4.23	0.882
	**Ferritin (ng/mL)**	1272.25	1362.59	654.85	480.20	433.16	438.79	565.06	633.16	497.72	0.000
	**Creatine phosphokinase (IU/L)**	311.64	747.16	350.86	171.08	347.19	220.33	221.54	537.01	275.37	0.019
	**Lactate dehydrogenase (U/L)**	880.96	698.83	714.63	247.76	271.10	246.54	475.06	471.72	443.93	0.000
	**IL-6 (pg/mL)**	131.55	58.05	121.56	15.54	42.43	220.94	79.99	52.29	159.42	0.788
	**C-reactive protein (mg/dL)**	158.35	155.15	137.60	74.62	50.50	37.65	106.02	101.97	79.61	0.000
	**Fibrinogen (mg/dL)**	502.78	584.49	504.14	450.55	488.62	449.89	469.30	533.69	471.99	0.001
	**Erythrocyte sedimentation rate (mm/h)**	53.71	64.53	42.10	39.04	35.39	29.96	40.84	41.66	33.76	0.000
	**WBCs (µL)**	27,195.15	22,990.80	24,083.28	9470.16	11,487.60	8455.86	16,253.80	17,239.20	14,935.52	0.000
	**Neutrophil-to-lymphocyte ratio**	16.77	21.68	15.80	6.18	6.05	7.42	10.23	13.86	10.89	0.000
	**Pulmonary CT-scan score**	16.32	16.80	13.97	6.08	6.61	5.25	10.00	11.67	8.93	0.000

**Table A17:** Distribution of patients across different risk categories of inflammation based on the WHO ordinal clinical severity scale.

		Risk Categories of Inflammation			Total
		Low	Intermediate	High	
**WHO ordinal clinical severity scale**	**3**	82	25	9	116
	**4**	32	9	8	49
	**5**	8	21	14	43
	**6**	0	28	15	43
	**7**	0	23	13	36
**Total**		122	106	59	287

**Table A18:** Cross-tabulation between WHO ordinal clinical severity scale and risk categories of inflammation by outcome.

Outcome			Risk Categories of Inflammation			Total	Sig.
			Low	Intermediate	High		
**Death**	**WHO ordinal clinical severity scale**	3	0	7	1	8	0.090
		4	1	2	5	8	
		5	1	18	13	32	
		6	0	28	15	43	
		7	0	23	13	36	
	**Total**		2	78	47	127	
**Alive**	**WHO ordinal clinical severity scale**	3	82	18	8	108	0.926
		4	31	7	3	41	
		5	7	3	1	11	
	**Total**		120	28	12	160	
**Total**	**WHO ordinal clinical severity scale**	3	82	25	9	116	<0.001
		4	32	9	8	49	
		5	8	21	14	43	
		6	0	28	15	43	
		7	0	23	13	36	
	Total		122	106	59	287	
